# Cardiopulmonary exercise testing and exercise recommendations for persons with anorexia nervosa

**DOI:** 10.1007/s40519-023-01591-y

**Published:** 2023-07-25

**Authors:** Solfrid Bratland-Sanda, Therese Fostervold Mathisen, Øyvind Støren

**Affiliations:** 1 Department of Sport, Physical Education and Outdoor Studies, University of South-Eastern Norway, Gullbringvegen 36, Bø, 3800 Telemark, Norway; 2 Faculty of Health, Welfare, and Organization, Østfold University College, Fredrikstad, Norway

**Keywords:** Fitness, Physical activity, Psychiatry

We want to address some concerns with the paper by Yamashita et al. [1]. We challenge the use and description of the rather old term anaerobic threshold (AT), in recent studies termed lactate threshold (LT), as an indicator of exercise tolerance. The statement that AT sets an upper limit for aerobic exercise intensity is incorrect. This upper limit is determined by maximal oxygen uptake (VO_2max_), a variable not influenced by AT/LT. The statement that the increase of acid levels above AT is unsafe is contextual and not justified for this paper’s clinical sample [i.e., persons with Anorexia Nervosa (AN) who are medically cleared to perform cardiopulmonary exercise testing]. This paper also lacks inclusion of the recent advances in exercise physiology advocating a different understanding of lactate metabolism during exercise [2].

The AT values presented in the paper [1] are remarkably low, questioning the validity of the data. AT corresponds to approximately 75–85% of VO_2max_, and this indicates a VO_2max_ of 12–15 mL kg^−1^⋅min^−1^ for the persons with AN and 17–20 mL⋅kg^−1^⋅min^−1^ for the healthy controls. This is below reference values for healthy 80 + years [3], and results are thus doubtful given the control group’s young age, normal body mass index and healthy condition. The results are probably based on inaccurate measurements and/or non-representative healthy controls. This paper lacks a thorough discussion of other factors potentially influencing the results. One such factor can be hyperventilation, which can explain the high respiratory frequency with low ventilation shown in the results [1]. We have ourselves experienced that this can occur during cardiopulmonary exercise testing among persons with AN. Weight regain is a core part of treatment for persons with AN, yet this paper does not present the variance of the weight regain during treatment, patients’ adherence to the imposed sedentary behavior, or discuss how ~ 3 months of imposed sedentary behavior with concurrent weight regain of ~ 5 kg might influence on the cardiopulmonary exercise test performed at discharge.

Based on the limitations with the design of the study and the collected data, there is a skew premise for the aim and an overinterpretation of the results. We acknowledge the need for regulating aerobic exercise in persons with AN, yet the existing reluctance among clinicians for using supervised and healthy exercise during treatment require high quality research. This paper, in our opinion, does not add knowledge to confirm or falsify existing myths in this field, and the findings are not suitable for making recommendations about exercise intensity for persons with AN.

## Data Availability

Not applicable.
